# Supplementary figures and images for: The Accuracy of the Uganda National Tuberculosis and Leprosy Program diagnostic algorithm and the World Health Organisation treatment decision algorithms for childhood tuberculosis: A retrospective analysis
Source: PLOS Glob Public Health. 2025 Apr 7;5(4):e0004026. doi: 10.1371/journal.pgph.0004026 (PMC11975100; doi:10.1371/journal.pgph.0004026)

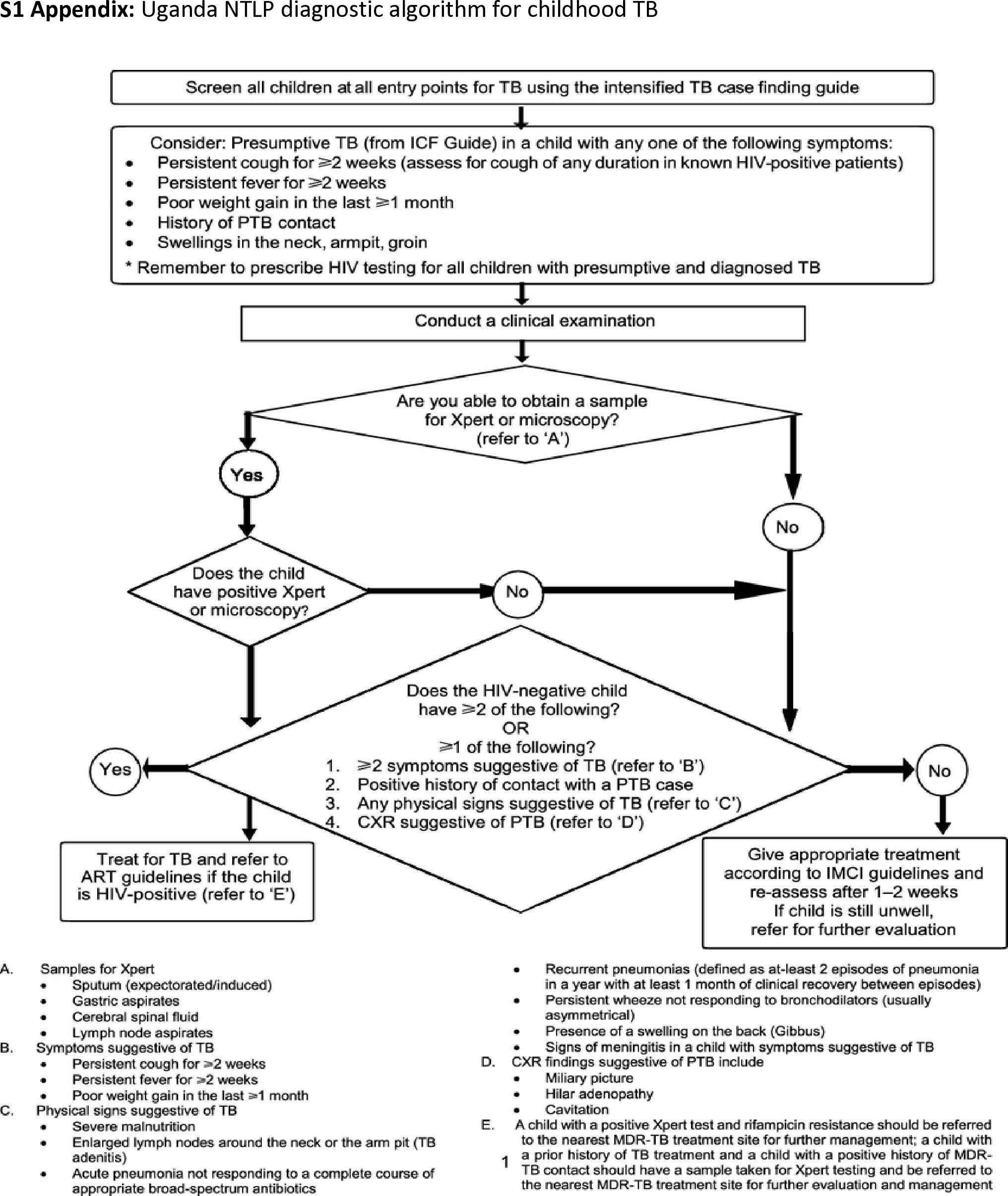

Supplement: S1 Appendix — A clinical decision tool designed by the Uganda national Tuberculosis and Leprosy Program (NTLP) to aid healthcare providers in diagnosing and managing TB in children. (TIF) [file pgph.0004026.s001.tif]

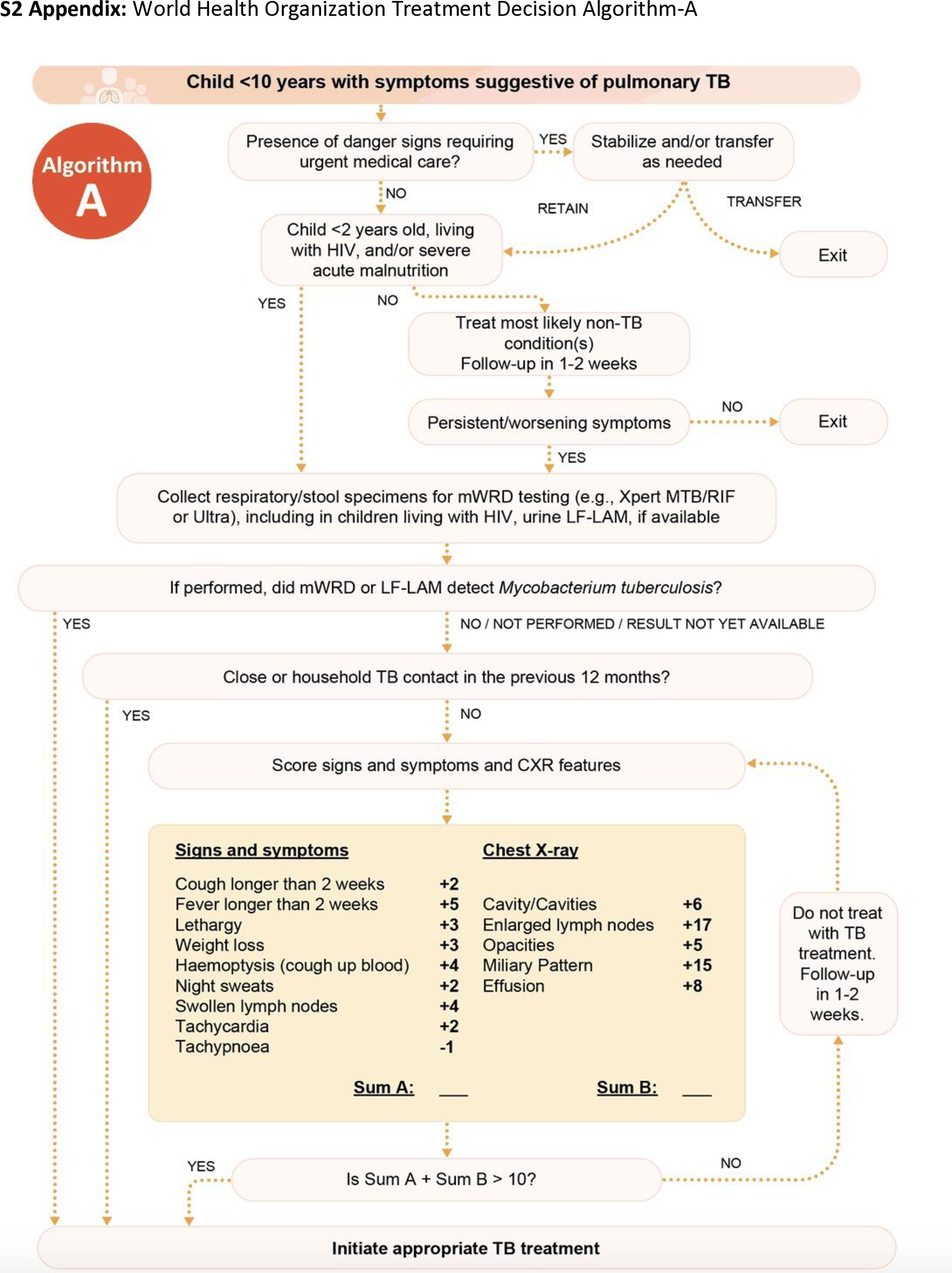

Supplement: S2 Appendix — Treatment decision algorithms (TDAs) revised in 2022 by the World Health Organisation to improve TB diagnosis in children in settings with chest x-ray. (TIF) [file pgph.0004026.s002.tif]

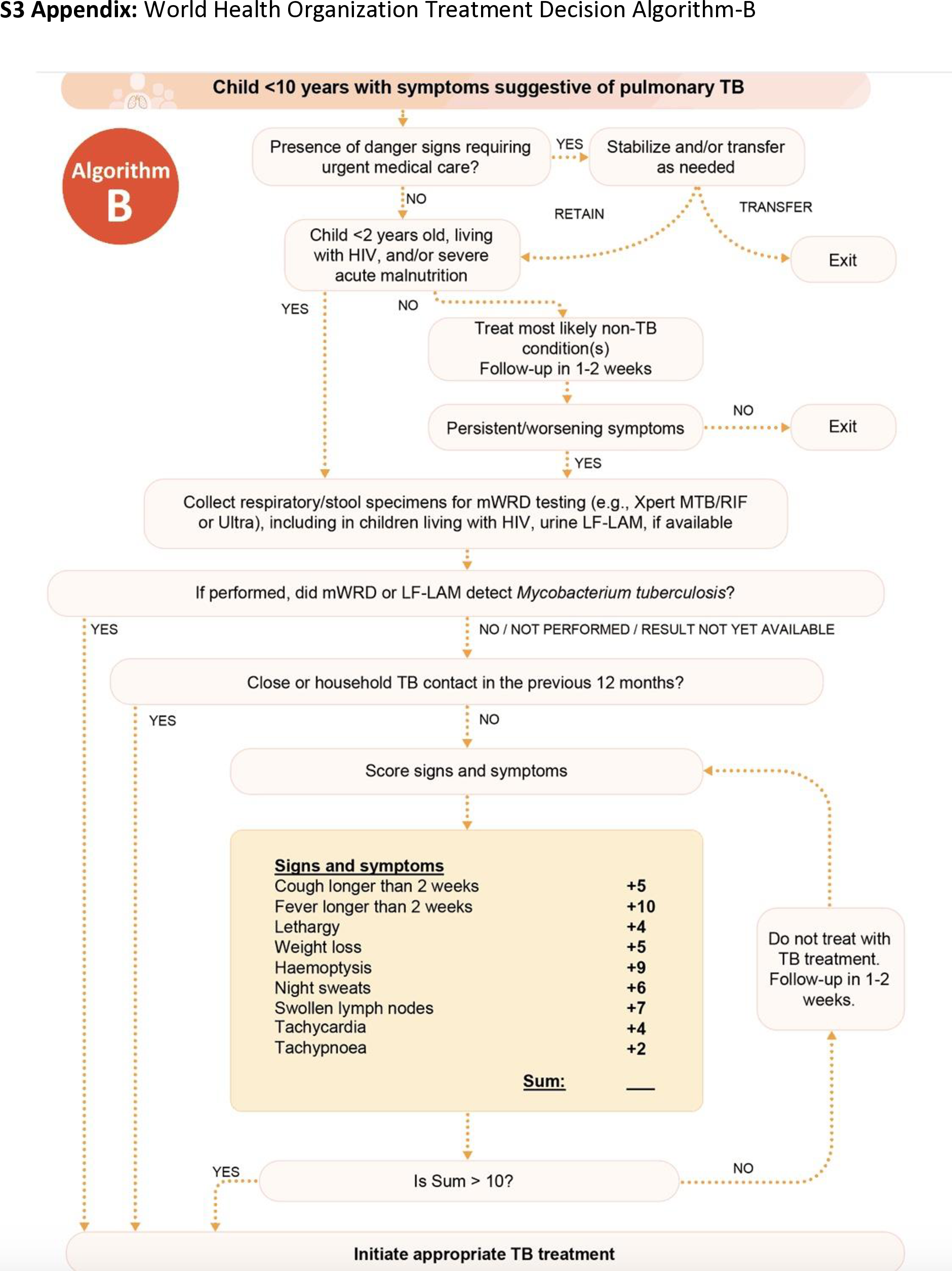

Supplement: S3 Appendix — Treatment decision algorithms (TDAs) revised in 2022 by the World Health Organisation to improve TB diagnosis in children in settings without chest x-ray. (TIF) [file pgph.0004026.s003.tif]

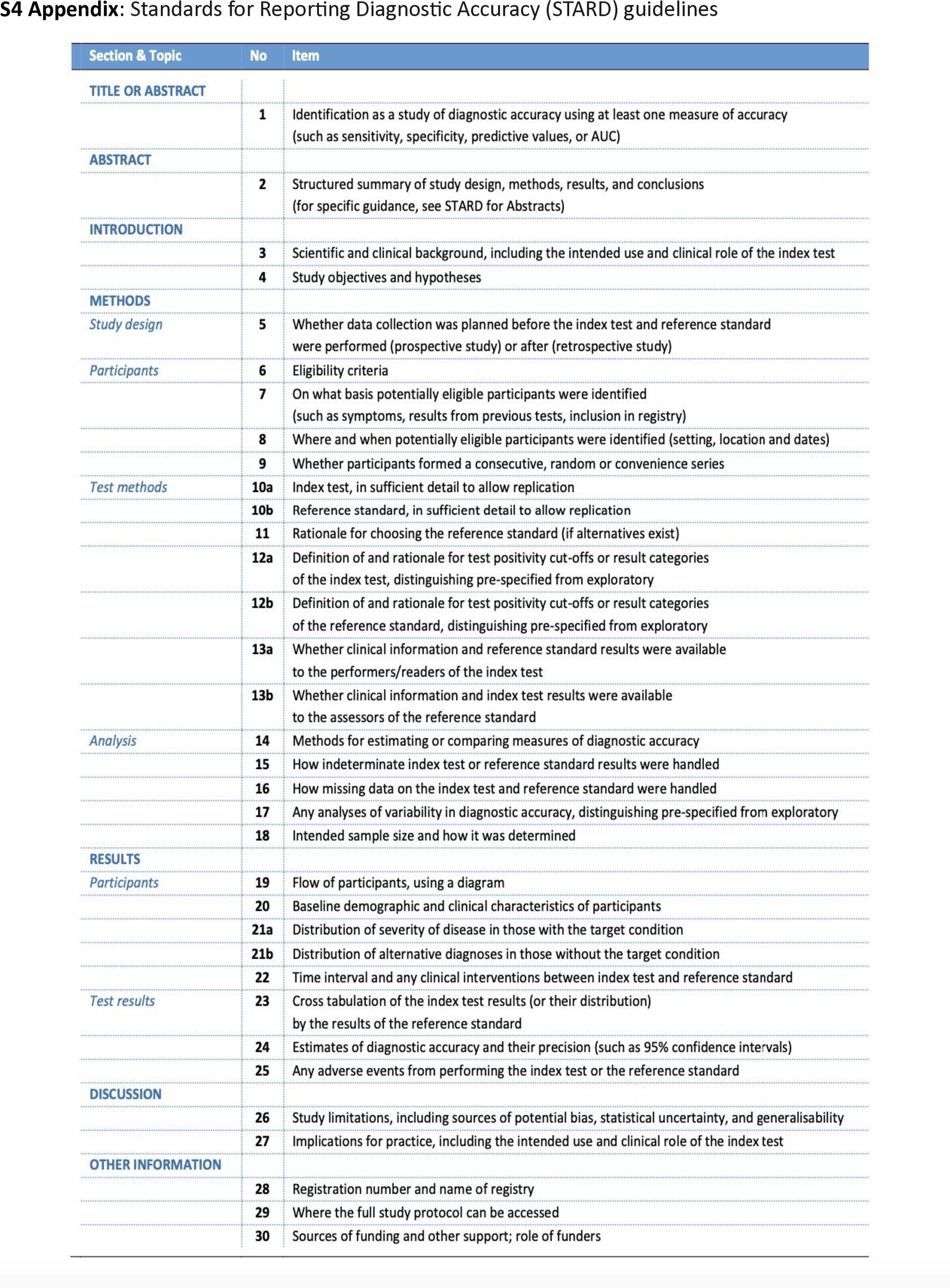

Supplement: S4 Appendix — Guidelines followed by this study to ensure that we provide sufficient details on methodology, execution, and results, allowing for a comprehensive assessment of our test’s validity and applicability. (TIF) [file pgph.0004026.s004.tif]
