# Supplementary material for: The Accuracy of the Uganda National Tuberculosis and Leprosy Program diagnostic algorithm and the World Health Organisation treatment decision algorithms for childhood tuberculosis: A retrospective analysis
Source: PLOS Glob Public Health. 2025 Apr 7;5(4):e0004026. doi: 10.1371/journal.pgph.0004026 (PMC11975100; doi:10.1371/journal.pgph.0004026)
Supplement: Attachment — Submitted filename: Academic Editor PLOS Global Health.docx [file pgph.0004026.s005.docx]

Academic Editor

PLOS Global Health

19^th^ December 2024

Dear Sir

**RE: PLOS Global Health 20241121**

Thanks for the opportunity to review PLOS Global Health 20241121. This paper attempts to add more knowledge to the knowledge base regards the use of Treatment Diagnostic Algorithms for Pediatric TB. Especially given that the current WHO Treatment Diagnostic Algorithm has a conditional recommendation and there is need for more evidence.

However, I have some comments, and they are written line by line below:

Generally, the authors should follow the guidance for writing Diagnostic Accuracy Tests, ***Bossuyt PM, Reitsma JB, Bruns DE, Gatsonis CA, Glasziou PP, Irwig L, LijmerJG Moher D, Rennie D, de Vet HCW, Kressel HY, Rifai N, Golub RM, Altman DG, Hooft L, Korevaar DA, Cohen JF, For the STARD Group. STARD 2015: An Updated List of Essential Items for Reporting Diagnostic Accuracy Studies.***

Line 1: It is important to clarify that this study is about the diagnostic accuracy of the Uganda National Childhood TB Algorithm and the WHO treatment algorithm for childhood Tuberculosis. Adding the words diagnostic and childhood TB helps with clarity.

Line 28: I would put a full stop after challenge. Then begin the next sentence with this has led, this helps with clarity. The sentence is shorter and easier to read.

Line 32: Add that the Uganda NTLP algorithm is for diagnosing childhood TB.

**INTRODUCTION**

Line 53-56: Could you give a mention to other samples that can be used to diagnose TB in children i.e stool. Which most people think would be easier to obtain.

Line 59: At the end of delays in treatment, there is need for a reference or citation.

Line 73: There is a need for a reference or citation.

**MATERIALS AND METHODS**

It may be important to start the methods, by clarifying what the study design is or was.

Line 81: You say the primary data. Was this a secondary data analysis. If yes, what was the primary use of the data.

Line 92-100: The criterion for the inclusion is not clear, because there are two criteria given one is the ICF and there is another described between line 92-96. Then line 96-100.

Line 116-line 120: could have been written better for clarity, please consider re-writing it.

Line 122-27: Please consider explain the WHO TDA better and also add both it and the Uganda National TB algorithm to the annexes.

**RESULTS**

The results could be better reported, there are other dimensions of diagnostic accuracy, apart from the sensitivity and specificity. These include negative predictive values, positive predictive values and there is also needed to provide raw data for the true positives, false positives, and true negatives and false negatives. Then you could also calculate the overall diagnostic accuracy of the test.

Line 188-190: Please write the results and refer to the Tables for further details not the other way around.

**DISCUSSION**

The discussion for diagnostic accuracy seeks to answer a few questions.

1. Can we use this test?
2. If we can use this test, when along the diagnostic process can we use it, is it for screening or diagnosis

Alternatively, you raised a few questions in lines 69-70. “Moreover, it is unclear if the new WHO algorithm should be utilized in Uganda, or the current Uganda NTLP algorithm should be continued or updated”. This paper doesn’t answer this question.

The Negative Predictive Value (NPV) and Positive Predictive Value (PPV) are sensitive to the prevalence of disease, so it is important to find out what happens to these values at 5%, 10% and 15% or 20%. This will further guide when these algorithms can be used.

Yours Sincerely

Michael Kakinda
