# Supplementary material for: The Accuracy of the Uganda National Tuberculosis and Leprosy Program diagnostic algorithm and the World Health Organisation treatment decision algorithms for childhood tuberculosis: A retrospective analysis
Source: PLOS Glob Public Health. 2025 Apr 7;5(4):e0004026. doi: 10.1371/journal.pgph.0004026 (PMC11975100; doi:10.1371/journal.pgph.0004026)
Supplement: Attachment — Submitted filename: Response to Reveiwers.docx [file pgph.0004026.s006.docx]

**Response to Reviewers**

**We thank the reviewers for their thoughtful feedback that has strengthened the work. Below is a point-by-point response:**

**Reviewer #1**

1. Generally, the authors should follow the guidance for writing Diagnostic Accuracy Tests, Bossuyt PM, Reitsma JB, Bruns DE, Gatsonis CA, Glasziou PP, Irwig L, LijmerJG Moher D, Rennie D, de Vet HCW, Kressel HY, Rifai N, Golub RM, Altman DG, Hooft L, Korevaar DA, Cohen JF, For the STARD Group. STARD 2015: An Updated List of Essential Items for Reporting Diagnostic Accuracy Studies.

**Response: We have reviewed the STARD checklist and updated the draft accordingly; we have added a completed checklist as an appendix [S1 Appendix], and cited the STARD criteria. Line 174**

2. Line 1: It is important to clarify that this study is about the diagnostic accuracy of the Uganda National Childhood TB Algorithm and the WHO treatment algorithm for childhood Tuberculosis. Adding the words diagnostic and childhood TB helps with clarity.

**Response: We have updated the title to reflect this guidance in Line 1**

3. Line 28: I would put a full stop after challenge. Then begin the next sentence with this has led, this helps with clarity. The sentence is shorter and easier to read.

**Response: Thank you for the guidance, this has been updated. Line 28**

4. Line 32: Add that the Uganda NTLP algorithm is for diagnosing childhood TB.

**Response: Thank you for the guidance, this has been updated. Line 32**

INTRODUCTION
5. Line 53-56: Could you give a mention to other samples that can be used to diagnose TB in children i.e stool. Which most people think would be easier to obtain.

**Response: We have added a statement in this regard especially about stool collection. Line 77-78.**

6. Line 59: At the end of delays in treatment, there is need for a reference or citation.

**Response: This has been updated. Line 83**

7. Line 73: There is a need for a reference or citation.

**Response: This has been updated. Line 89**

MATERIALS AND METHODS
8. It may be important to start the methods, by clarifying what the study design is or was.
**Response: We have updated this to emphasize our secondary analysis. Line 118**

9. Line 81: You say the primary data. Was this a secondary data analysis. If yes, what was the primary use of the data.

**Response: We have clarified that we did secondary data analysis and the aim of the parent study to read “The parent study, whose main aim was to evaluate performance of novel diagnostic tests, was situated at the Mulago National Referral Hospital Pediatric TB clinic within the Mulago National Referral Hospital (MNRH). Line 118-120**

10. Line 92-100: The criterion for the inclusion is not clear, because there are two criteria given one is the ICF and there is another described between line 92-96. Then line 96-100.

**Response: We have included a sentence to show what the inclusion criteria was for the parent study Lines 130-135 versus for our analysis which was restricted to children who met the Intensified Case Finding (ICF) criteria. Line 135-149**

11. Line 116-line 120: could have been written better for clarity, please consider re-writing it.

**Response: Thank you for the guidance, we have made the changes to make the statements clearer. Line 164-169**

12. Line 122-27: Please consider explain the WHO TDA better and also add both it and the Uganda National TB algorithm to the annexes.

**Response: We have added Appendices with references to schematically describe the TDAs Line 174 and 180**

RESULTS
13. The results could be better reported, there are other dimensions of diagnostic accuracy, apart from the sensitivity and specificity. These include negative predictive values, positive predictive values and there is also needed to provide raw data for the true positives, false positives, and true negatives and false negatives. Then you could also calculate the overall diagnostic accuracy of the test.

**Response: We have added to Table 3 NPV and PPV under three prevalences in Table 3.**

14. Line 188-190: Please write the results and refer to the Tables for further details not the other way around.

**Response: We have updated Line 257 as well as Line 259 to summarize the results and refer to the tables for more details.**

DISCUSSION
15. The discussion for diagnostic accuracy seeks to answer a few questions.
1. Can we use this test?
2. If we can use this test, when along the diagnostic process can we use it, is it for screening or diagnosis
Alternatively, you raised a few questions in lines 69-70. “Moreover, it is unclear if the new WHO algorithm should be utilized in Uganda, or the current Uganda NTLP algorithm should be continued or updated”. This paper doesn’t answer this question.
The Negative Predictive Value (NPV) and Positive Predictive Value (PPV) are sensitive to the prevalence of disease, so it is important to find out what happens to these values at 5%, 10% and 15% or 20%. This will further guide when these algorithms can be used.

**Response: We have made changes to the discussion to show utility of the algorithm in its current form. The high sensitivity supports their goal to ensure there are few missed cases, which is a major challenge in childhood TB care. At the same time, the low specificity suggests that this will come at a cost of overtreatment, as noted in Lines 340-342. We have added a new Table 3 to show the PPV and NPV at TB prevalence of 5%, 12% and 20%, and noted in lines 397-398 that the benefits of the TDAs are greater in high TB prevalence settings.**

**Reviewer #2**

1. Line 36: When referring to the NIH definition, it may be useful to indicate the main classifications (confirmed, unconfirmed, unlikely TB)

**Response: We have revised this section to include the consensus definitions; including Confirmed, Unconfirmed and Unlikely TB classification. Line 37**

2. Line 45: For enhanced understanding it may be good to provide a definition of high-risk children (high risk of rapid disease progression/severe disease/something else?)

**Response: We have defined high-risk under risk group categorization. We based this on their higher risk of rapid disease progression, as defined by the WHO. Line 212-213**

3. Line 61: there is a reference to the 2021 Global Tuberculosis report, however, 5-year progress against the 2018 UN HLM targets was presented in the 2023 report and the % was 71% (the 41% was an interim figure after only 3 years so this should not be used in this context). It may be better to use treatment coverage as an indicator here rather than progress against the UN HLM target. In 2023 (as reported in the 2024 Global TB report) treatment coverage was 55% for the age group <15 years (but only 48% in the age group below 5 years).

**Response: We have revised this line to read “treatment coverage was only 55% for children < 15 years (and even lower for the age group below 5 years at 48%) according to the Global TB report of 2024”. We updated the reference accordingly. Line 81-83**

4. Line 67: please clarify that the TDAs are included in the WHO operational handbook and that the recommendation in the guidelines is on TDAs in general (not for any algorithm in particular).

**Response: We have added a sentence to read ‘’ In 2022, the World Health Organization (WHO) conditionally recommended the use of TDAs for childhood TB, and presented (in its operational handbook) an algorithm based on signs and symptoms as well as scores with CXR (Algorithm A) or without CXR (Algorithm B).’’ Lines 87-89**

5. Line 67-69: the main issue to note is that the TDAs in the WHO operational handbook have only been internally validated and WHO has called for external validation of the algorithms.

**Response: We have added a sentence to read “However, these TDAs have only been internally validated, and therefore the WHO has called for their external validation.’’ Line 89-90**

6. Study population: it will also be important to note (in the discussion) that the WHO TDAs are designed for use at lower levels of the health care system, and conducting a retrospective analysis of tertiary level data may introduce bias (this needs to be included in the discussion under limitations).

**Response: In this study, children were also referred from lower levels of health care. This has further been clarified in the discussion, while also acknowledging the potential bias of its use in higher levels of care. Lines 405-407**

7. Line 114: it may be good to include the NTLP algorithm/decision tree and the WHO TDAs as images to clarify the steps if possible.

**Response: Following this guidance, we have clarified both NTLP algorithm and WHO TDAs in Lines 165-174. We have further added the decision tree for both as Appendices S2 and S3 respectively.**

8. Lines 122-127: an important omission in the description of the WHO TDAs is the steps that are included before molecular testing is done: these include danger signs and relevant urgent management and also an assessment of risk of rapid disease progression, whereby children at high risk of rapid disease progression (those aged <2 years, those with HIV infection or those with SAM) immediately continue through the algorithms, but children at low risk of rapid disease progression are first treated for alternative diagnoses and only return for the next steps if they have not improved after this treatment. In a retrospective study, it will be very difficult to correctly evaluate this step as this data may not be available. This is a major limitation of this study and this needs to be acknowledged in the discussion. It is not clear from the description of the methodology which steps of the WHO TDAs were taken into account, this will need to be further clarified. It seems that only the results of molecular testing and the scoring sections were considered.

**Response: We have discussed that due to our retrospective study design, it was difficult to make prospective assessments of follow up of low-risk children. This has been acknowledged as a limitation. Lines 407- 410**

9. Line 149: for the overall…. It seems there is a word missing

**Response: We have added ‘estimate’ under Line 212. Thank you for the guidance**

10. Line 228-229: this statement suggests that the only part that was taken into account was the scoring section of the WHO TDAs, but not the additional steps (danger signs and risk assessment) that were added in an attempt to improve accuracy (notably specificity). It suggests therefore that the retrospective methodology is not adequate to assess these algorithms, and a prospective design would be needed to evaluate the algorithms comprehensively. It will be important to highlight this as a limitation in the discussion.

**Response: We have added a sentence under limitations to read ‘The WHO TDAs incorporates additional steps including danger signs in an attempt to improve specificity; these are difficult to assess in a retrospective study design’’. Of note, the WHO TDA approach is different from how the Uganda NTLP algorithm is implemented (for all persons who have presumptive TB at the time of assessment), and we sought to compare it (WHO TDAs) to the current standard of care in Uganda. Lines 406-411**

11. Lines 239-240: it may be good to explain why the score in the WHO algorithm differs – and reference the Gunasekera paper here (currently ref 12)

**Response: According to Gunasekera et al, models that included chest x-ray had high specificity, similar to our findings (Table 2). Our explanation is in the way these scores are weighted, including different weights for the x-ray findings. This is unlike the Uganda NTLP algorithm. Line 359 – 366.**

12. From line 252 on limitations: here the additional limitations mentioned above (mainly related to disregarding the additional steps in the WHO algorithms) need to be included. It may be good to mention that an operational research study is currently being implemented to prospectively evaluate the WHO TDAs in various settings in Uganda. In addition, it will be important to check and if so, to state, if a subset of the data reviewed here in this dataset was used for the development of the WHOP TDAs (see also the Gunasekera paper).

**Response: We have added a section under study strengths to read ‘The data was not used to develop the WHO TDAs, and so will be valuable for the external validation.’ Lines 393-394**

13. Lines 256-257: in addition, the algorithms (both the NTLP and WHO ones) are designed to be used at PHC level, where the prevalence of TB may be lower and where children generally present with less severe forms of TB, and where capacity to conduct Xpert testing/specimen collection and CXR may be limited and thus overdiagnosis may be an important issue in these settings. Under Lines 269-270

**Response: We agree with the reviewer’s addition. We have raised similar concerns in Lines 395 – 398.**

14. From line 261: it will be important to flag here the limitations of the retrospective methodology as mentioned before. Capacity building and regular mentorship will be other important aspects to take into consideration.

**Response: We have through this resubmission highlighted several limitations of our study ranging from bias associated with the reference to the study design. Lines 398 – 435. We however add much needed knowledge on the accuracy of the algorithms.**

15. References: need substantial editing as many authors/organizations are not reflected correctly.

**Response: Thank you for the guidance. We have edited and corrected the references following the Vancouver referencing standard. Lines 450- 492**
